# High rates of meticillin-resistant *Staphylococcus aureus* among asylum seekers and refugees admitted to Helsinki University Hospital, 2010 to 2017

**DOI:** 10.2807/1560-7917.ES.2018.23.45.1700797

**Published:** 2018-11-08

**Authors:** Tuomas Aro, Anu Kantele

**Affiliations:** 1 Department of Internal Medicine, Clinicum, Medical Faculty, University of Helsinki, Helsinki, Finland; 2 Inflammation Center, University of Helsinki and Helsinki University Hospital, Helsinki, Finland; 3 Karolinska Institutet, Stockholm, Sweden

**Keywords:** refugee, asylum seeker, multi-drug resistance, antimicrobial resistance, AMR, ESBL, MRSA, Helsinki

## Abstract

**Introduction:**

Antimicrobial resistance is increasing rapidly in countries with low hygiene levels and poorly controlled antimicrobial use. The spread of resistant bacteria poses a threat to healthcare worldwide. Refugees and migrants from high-prevalence countries may add to a rise in multidrug-resistant (MDR) bacteria in low-prevalence countries. However, respective data are scarce.

**Methods:**

We retrospectively collected microbiological and clinical data from asylum seekers and refugees treated at Helsinki University Hospital between January 2010 and August 2017.

**Results:**

Of 447 asylum seekers and refugees (Iraq: 46.5%; Afghanistan: 10.3%; Syria: 9.6%, Somalia: 6.9%); 45.0% were colonised by MDR bacteria: 32.9% had extended-spectrum beta-lactamase-producing Enterobacteriaceae (ESBL-PE), 21.3% meticillin-resistant *Staphylococcus aureus* (MRSA), 0.7% carbapenemase-producing Enterobacteriaceae (CPE), 0.4% multiresistant *Pseudomonas aeruginosa* (MRPA), 0.4% multiresistant *Acinetobacter baumannii* (MRAB); no vancomycin-resistant *Enterococcus* (VRE) were found. Two or more MDR bacteria strains were recorded for 12.5% of patients. Multivariable analysis revealed geographical region and prior surgery outside Nordic countries as risk factors of MRSA colonisation. Young age (< 6 years old), short time from arrival to first sample, and prior hospitalisation outside Nordic countries were risk factors of ESBL-PE colonisation.

**Conclusion:**

We found MDR bacterial colonisation to be common among asylum seekers and refugees arriving from current conflict zones. In particular we found a high prevalence of MRSA. Refugees and migrants should, therefore, be included among risk populations requiring MDR screening and infection control measures at hospitals.

## Introduction

Antimicrobial resistance (AMR), a major health problem worldwide, surges most rapidly in regions with low level of hygiene and poor control of antimicrobial use [1]. AMR spreads across the globe and its extent has been recognised by international bodies at the highest level: in 2016 AMR was addressed at a General Assembly session of the United Nations as the greatest and most urgent global health risk [2].

A major concern about AMR is its spread to healthcare settings in low-prevalence countries with severe consequences: treatment failures, increase in the number of serious infections, and dramatic cost implications [3]. To prevent the spread of AMR to hospitals, patients with particular risk of colonisation and infection with multidrug-resistant (MDR) bacteria should be identified and subjected to infection control measures at the admission stage. Numerous studies have identified international travel as a major risk factor for colonisation: approximately one third of visitors to high-prevalence regions acquire MDR bacteria during ordinary tourist travel [4-18]. Refugees and migrants who have lived for years in high-risk regions are presumed to have even higher levels of colonisation.

The European migrant crisis began in 2015 when over 1.2 million first-time asylum seekers (this group includes both refugees and migrants) applied for international protection in European Union countries [19,20]. Finland received 32,476 applications for asylum in 2015, an almost 10-fold increase on previous years [21].

Like international travel, migration may contribute substantially to the spread of AMR [22-26]. Refugees and migrants mostly come from countries with considerably higher rates of MDR bacteria than Finland and, moreover, they may have journeyed through other high-prevalence regions [1]. Accurate data on colonisation rates in this population are required to estimate transmission risk and to prepare infection control guidelines for hospitals. This retrospective study investigates the prevalence of various MDR bacteria among asylum seekers and refugees hospitalised in Finland, and seeks risk factors that can be used to identify those at highest risk of colonisation.

## Methods

### Selection of participants

Helsinki University Hospital (HUCH) provides secondary and tertiary care for the 1.6 million inhabitants of southern Finland. During the study period, from January 2010 to August 2017, our hospital’s infection control guidelines stated that all asylum seekers and refugees admitted to hospitals should be screened at entry for meticillin-resistant *Staphylococcus aureus* (MRSA), vancomycin-resistant *Enterococcus* (VRE), extended-spectrum beta-lactamase-producing Enterobacteriaceae (ESBL-PE), carbapenemase-producing Enterobacteriaceae (CPE), multiresistant *Acinetobacter baumannii* (MRAB) and multiresistant *Pseudomonas aeruginosa* (MRPA). The same screening guidelines were applied to patients who had been hospitalised outside the Nordic countries during the previous 12 months before admission to our hospital.

**Figure 1:**
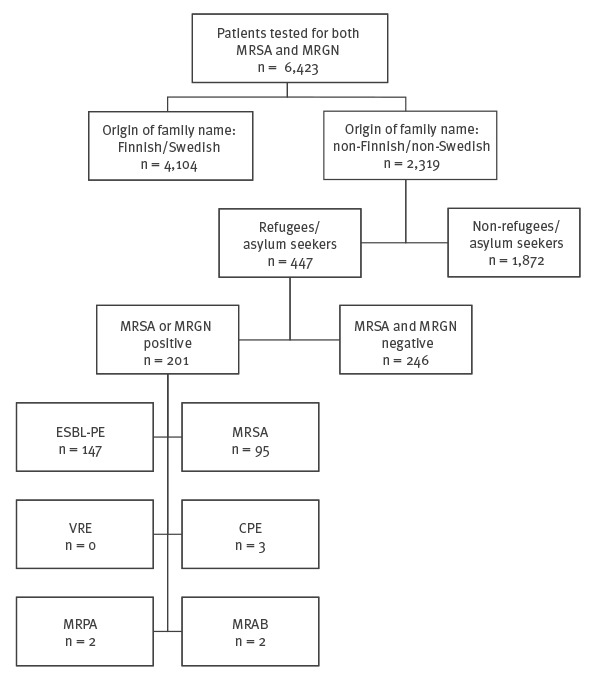
Flowchart showing multidrug-resistant bacteria found in samples from asylum seekers and refugees admitted to Helsinki University Hospital, Finland, January 2010 to August 2017 (n = 447)

CPE: carbapenemase-producing Enterobacteriaceae; ESBL-PE: extended-spectrum beta-lactamase-producing Enterobacteriaceae; MRAB: multiresistant *Acinetobacter baumannii*; MRGN: multiresistant Gram-negative bacteria; MRPA: multiresistant *Pseudomonas aeruginosa*; MRSA: meticillin-resistant *Staphylococcus aureus*; VRE: vancomycin-resistant *Enterococcus*.

Using the HUCH infectious diseases database, SAI, we compiled a list of patients who had been sampled for both MRSA and multiresistant Gram-negative (MRGN) bacteria at hospital admission. Among these, we selected those with a non-Finnish/non-Swedish name and, after screening their patient charts, included only those who were asylum seekers or refugees (Figure 1). According to the Finnish Medical Research Act, review by an ethics committee is only required for research involving an intervention. The study protocol was approved by the research board of the Inflammation Center, Helsinki University Hospital, Finland.

### Collection of patient data

**Figure 2:**
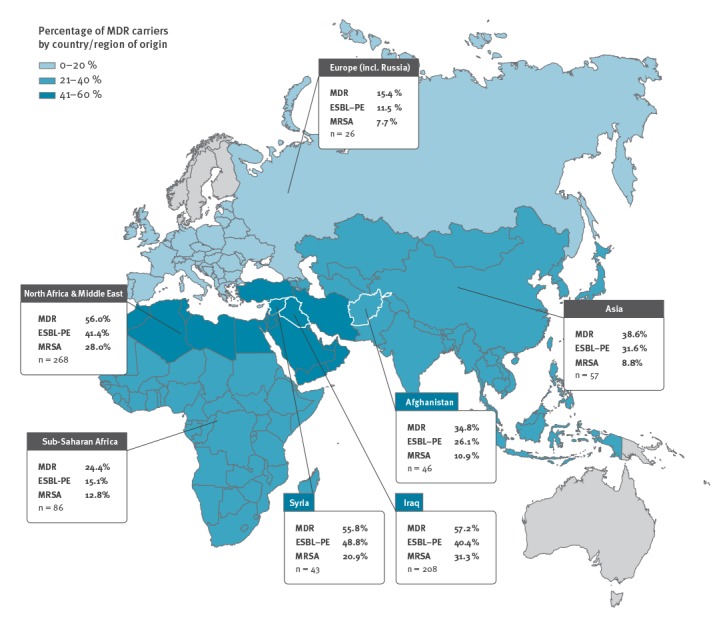
Rate of multidrug-resistant bacteria among asylum seekers and refugees admitted to Helsinki University Hospital, by country of origin or geographical region, Finland, January 2010 to August 2017 (n = 447)

ESBL-PE: extended-spectrum beta-lactamase-producing Enterobacteriaceae; MDR: multidrug-resistant bacteria; MRSA: meticillin-resistant *Staphylococcus aureus*.

For background information, we collected data from the patient records on sex, age, country of origin, date of arrival in Finland, prior hospitalisation and surgery (as recorded by the clinician), and determined Charlson Comorbidity Index (CCI) [27] for each subject. In addition, we collected data covering the results of bacterial cultures (blood, urine, stool), reason for admission, clinical diagnosis (ICD-10) [28] at discharge, MDR bacterial infections identified, and deaths. For further analysis, patients were grouped by geographical region according to their country of origin. Here we applied a classification (Figure 2) modified from United Nations geoscheme (Europe, North Africa and Middle East, sub-Saharan Africa, Asia, other) [29].

For nine patients, country of origin was not known. One patient was from Mexico (not shown in the map).

Patients’ countries of origin in the various geographical regions:

Europe: Albania, Georgia, Greece, Kosovo*, Russia, Serbia, Ukraine;

North Africa and the Middle East: Iraq, Iran, Jordan, Kuwait, Lebanon, Morocco, Syria, Turkey, Yemen;

Sub-Saharan Africa: Burundi, Cameroon, Congo, Ethiopia, Eritrea, Gambia, Ghana, Kenya, Nigeria, Rwanda, Somalia, South Africa, Sudan, Uganda, Zambia;

Asia: Afghanistan, Azerbaijan, Kazakhstan, China, Kyrgyzstan, Myanmar, Nepal, Pakistan, Sri Lanka.

*This designation is without prejudice to positions on status, and is in line with United Nations Security Council Resolution 1244/99 and the International Court of Justice Opinion on the Kosovo Declaration of Independence.

### Microbiological methods

According to the hospital guidelines, swabs for screening patients for MDR bacteria are collected as follows: MRSA samples are taken each with a separate swab from the nostrils (one swab for both), pharynx and rectum or perineum. MRGN bacteria samples are collected as rectal swabs. In addition, swabs are taken from wound infections when applicable. The screening comprises two sets of samples, and where possible the samples are to be collected on consecutive days.

While some minor modifications to the routine laboratory practices took place during the study period, at the time of the last sampling, MDR bacterial analyses were carried out as follows: MRSA was screened by overnight enrichment on eMRSA broth (Copan Italia, Brescia, Italy) or selective in-house MRSA enrichment broth [30] followed by culture on CHROMagar MRSA (CHROMagar, Paris, France), and confirmed with *S. aureus*-specific nuclease and mecA gene qPCR [30]. VRE were screened by enrichment Enterococcosel Broth (BBL, Cockeysville, MD, United States of America (USA)) and followed by culture on CHROMagar VRE media. Positive findings were confirmed by in-house PCR as described by Suppola et al. [31].

ESBL-PE and CPE were analysed by plating directly on CHROMagar ESBL and CHROMagar KPC or CHROMagar mSuperCARBA, respectively. ESBL-PE species identification was confirmed by MALDI-TOF (Vitek-MS, bioMérieux, Marcy l’Étoile, France) and resistance by standard EUCAST method [32]. CPE species were confirmed with in-house carbapenemase gene PCR.

MDR-*P. aeruginosa* and MDR-*A. baumannii* were screened from ESBL and KPC SuperCARBA plates. Cultures were tested by MALDI-TOF for species identification. Isolates resistant to meropenem for *Acinetobacter*, and both meropenem and ceftazidime for *Pseudomonas*, were analysed by PCR for carbapenemase genes as previously described [33].

### Statistics

Data were entered on Microsoft Excel 2013 spreadsheets, and statistical analyses were conducted using SPSS 24.0.0.0 software (IBM Corp., Armonk, NY, USA). In univariate analyses for categorical variables, chi-squared test, Fisher’s exact test or binary logistic regression analysis was applied. For continuous variables, we used binary logistic regression. Chi-squared test and Fisher’s exact test were two-sided. Variables for the multivariable model were selected using the p value limit of 0.2 in the univariate model. Time between arrival and first sample was not known for all cases; missing values were taken into account by multiple imputations, assuming that data were missing at random. In the multivariable model, we used backward selection with Akaike information criteria (AIC) so as to choose the most informative explanatory variables for the final model. From several highly correlated variables, only one was included.

## Results

### Subject characteristics

According to the HUCH infectious diseases database SAI, 6,423 patients were screened for both MRSA and MRGN bacteria at admission between 1 January 2010 and 23 August 2017. A total of 2,319 patients with a non-Finnish/non-Swedish name were selected and their patient records screened (Figure 1). The final study population included 447 patients with refugee or asylum seeker status stated in their patient records. The increase in the number of refugees and asylum seekers admitted during the study period was evident: the vast majority (86.8%) were hospitalised after the beginning of 2015. As shown by the demographic data presented in Tables 1 and 2, their median age was 25 years, and 53.0% were female. The patients had 41 different countries of origin, the majority in Iraq (46.5%), Afghanistan (10.3%), Syria (9.6%), and Somalia (6.9%). Most of the subjects (93.3%) were healthy, with a CCI-score of 0. Prior hospitalisation outside the Nordic countries was recorded for 17.9%, an invasive procedure for 11.0%, and treatment in an intensive care unit abroad for 4.0% of the patients. The median time from arrival in Finland to first MDR bacterial sampling was 59 days. The date of arrival in Finland was available for 283/447 patients (63.3%).

**Table 1:** Background characteristics of asylum seekers and refugees admitted to Helsinki University Hospital, by geographical region of origin^a^, Finland, January 2010–August 2017 (n = 447 patients)

Patient attributes	Europe n = 26				North Africa and Middle East n = 268		Sub-Saharan Africa n = 86		Asia n = 57		Other or unknown n = 10		Total n = 447	
	n		%		n	%	n	%	n	%	n	%	n	%
**Sex**														
Male	8		30.8		135	50.4	32	37.2	31	54.4	4	40.0	210	47.0
Female	18		69.2		133	49.6	54	62.8	26	45.6	6	60.0	237	53.0
Median age (years)	24				26		27		20		27		25	
**Age group (years)**														
0–5	3			11.5	36	13.4	7	8.1	6	10.5	2	20.0	54	12.1
6–15	3			11.5	32	11.9	3	3.5	15	26.3	0	0.0	53	11.9
16–25	9			34.6	64	23.9	31	36.0	14	24.6	3	30.0	121	27.1
26–35	8			30.8	98	36.6	30	34.9	12	21.1	4	40.0	152	34.0
> 35	3			11.5	38	14.2	15	17.4	10	17.5	1	10.0	67	15.0
**Charlson Comorbidity Index**														
0 points	24			92.3	251	93.7	80	93.0	52	91.2	10	100.0	417	93.3
1 point	0			0.0	3	1.1	1	1.2	2	3.5	0	0.0	6	1.3
2–3 points	2			7.7	11	4.1	4	4.7	3	5.3	0	0.0	20	4.5
4–5 points	0			0.0	1	0.4	1	1.2	0	0.0	0	0.0	2	0.4
> 5 points	0			0.0	2	0.7	0	0.0	0	0.0	0	0.0	2	0.4
**Mean time from arrival to Finland to first sample (days)**	**78**				**127**		**100**		**119**		**363**		**119**	
**Prior treatment history**														
Prior hospitalisation abroad	5	19.2			54	20.1	15	17.4	6	10.5	0	0.0	80	17.9
Prior invasive procedure abroad	2	7.7			34	12.7	9	10.5	4	7.0	0	0.0	49	11.0

^a^Patients’ countries of origin in the various geographical regions:

Europe: Albania, Georgia, Greece, Kosovo*, Russia, Serbia, Ukraine;

North Africa and Middle East: Iraq, Iran, Jordan, Kuwait, Lebanon, Morocco, Syria, Turkey, Yemen;

Sub-Saharan Africa: Burundi, Cameroon, Congo, Ethiopia, Eritrea, Gambia, Ghana, Kenya, Nigeria, Rwanda, Somalia, South Africa, Sudan, Uganda, Zambia;

Asia: Afghanistan, Azerbaijan, Kazakhstan, China, Kyrgyzstan, Myanmar, Nepal, Pakistan, Sri Lanka;

Other or unknown: Mexico, unknown.

*This designation is without prejudice to positions on status, and is in line with United Nations Security Council Resolution 1244/99 and the International Court of Justice Opinion on the Kosovo Declaration of Independence.

**Table 2:** Background characteristics of asylum seekers and refugees admitted to Helsinki University Hospital, by country of origin^a^, Finland, January 2010–August 2017 (n = 447 patients)

Patient attributes	Iraq n = 208		Afghanistan n = 46		Syria n = 43		Somalia n = 31		Nigeria n = 16		Other or unknown n = 103		Total n = 447	
	n	%	n	%	n	%	n	%	n	%	n	%	n	%
**Sex**														
Male	109	52.4	25	54.3	19	44.2	11	35.5	4	25.0	42	40.8	210	47.0
Female	99	47.6	21	45.7	24	55.8	20	64.5	12	75.0	61	59.2	237	53.0
**Median age (years)**	**26**		**19**		**25**		**22**		**30**		**26**		**25**	
**Age group (years)**														
0–5	27	13.0	4	8.7	5	11.6	1	3.2	1	6.3	16	15.5	54	12.1
6–15	20	9.6	12	26.1	9	20.9	2	6.5	0	0.0	10	9.7	53	11.9
16–25	54	26.0	13	28.3	9	20.9	17	54.8	4	25.0	24	23.3	121	27.1
26–35	76	36.5	10	21.7	15	34.9	8	25.8	10	62.5	33	32.0	152	34.0
> 35	31	14.9	7	15.2	5	11.6	3	9.7	1	6.3	20	19.4	67	15.0
**Charlson Comorbidity Index**														
0 points	197	94.7	43	93.5	38	88.4	28	90.3	16	100.0	95	92.2	417	93.3
1 point	1	0.5	2	4.3	1	2.3	1	3.2	0	0.0	1	1.0	6	1.3
2–3 points	7	3.4	1	2.2	4	9.3	2	6.5	0	0.0	6	5.8	20	4.5
4–5 points	1	0.5	0	0.0	0	0.0	0	0.0	0	0.0	1	1.0	2	0.4
> 5 points	2	1.0	0	0.0	0	0.0	0	0.0	0	0.0	0	0.0	2	0.4

^a^The data are only presented for the five most common countries (in total, the study population originated in 41 different countries).

### Cause of hospitalisation

An infectious disease was the primary diagnosis for 83/447 patients (18.6%), most commonly confined to the respiratory (21.7%; 18/83) or urogenital tract (13.3%; 11/83), or presented as dermatological infection (16.9%; 14/83) or acute gastroenteritis (7.2%; 6/83). The majority of patients (81.4%) had a non-infectious disease and nearly one third (29.8%; 133/447) of the admissions were associated with pregnancy.

### Findings of multidrug-resistant bacteria

Almost half of the patients (45.0%; 201/447) were colonised by MDR bacteria: 32.9% had ESBL-PE, 21.3% MRSA, 0.7% CPE, 0.4% MRPA and 0.4% MRAB (Table 3). No cases with VRE were recorded. Two or more MDR strains were found in 12.5%. The proportion of MDR bacterial carriers was found greatest among the patients from Iraq and Syria (57.2% and 55.8% respectively), followed by Afghanistan (34.8%) and Somalia (25.8%) (Table 4). Analysis by geographical region (Table 3) showed the highest MDR bacterial colonisation rates for patients from North Africa and the Middle East (56.0%). Patients from Asia and sub-Saharan Africa had MDR bacterial carriage rates of 38.6% and 24.4%, respectively, while the result for those from Europe was 15.4% (Figure 2).

**Table 3:** The number of carriers of multidrug-resistant bacteria among asylum seekers and refugees admitted to Helsinki University Hospital, by geographical region of origin^a^, Finland, January 2010-August 2017 (n = 447 patients)

Patient attributes	Europe n = 26		North Africa and Middle-East n = 268		Sub-Saharan Africa n = 86		Asia n = 57		Other or unknown n = 10		Total n = 447^b^	
	n	%	n	%	n	%	n	%	n	%	n	%
Any MDR bacteria	4	15.4	150	56.0	21	24.4	22	38.6	4	40.0	201	45.0
MRSA	2	7.7	75	28.0	11	12.8	5	8.8	2	20.0	95	21.3
ESBL-PE	3	11.5	111	41.4	13	15.1	18	31.6	2	20.0	147	32.9
VRE	0	0.0	0	0.0	0	0.0	0	0.0	0	0.0	0	0.0
MRAB	0	0.0	1	0.4	1	1.2	0	0.0	0	0.0	2	0.4
MRPA	1	3.8	1	0.4	0	0.0	0	0.0	0	0.0	2	0.4
CPE	0	0.0	3	1.1	0	0.0	0	0.0	0	0.0	3	0.7
Multiple MDR bacteria ≥ 2 classes or strains of MDR bacteria	2	7.7	48	17.9	5	5.8	1	1.8	0	0.0	56	12.5
Clinical infection with MDR bacteria	0	0.0	8	3.0	1	1.2	1	1.8	0	0.0	10	2.2
Proportion of MDR bacteria carriers with clinical MDR bacterial infection (%)	0		5.3		4.8		4.5		0		5.0	

CPE: Carbapenemase-producing Enterobacteriaceae; ESBL-PE: extended-spectrum beta-lactamase-producing Enterobacteriaceae; MDR: multidrug-resistant; MRAB: multiresistant *Acinetobacter baumannii*; MRPA: multiresistant *Pseudomonas aeruginosa*; MRSA: meticillin-resistant *Staphylococcus aureus*; VRE: Vancomycin-resistant *Enterococcus*.

^a^Patients’ countries of origin in the various geographical regions:

Europe: Albania, Georgia, Greece, Kosovo*, Russia, Serbia, Ukraine;

North Africa and Middle East: Iraq, Iran, Jordan, Kuwait, Lebanon, Morocco, Syria, Turkey, Yemen;

Sub-Saharan Africa: Burundi, Cameroon, Congo, Ethiopia, Eritrea, Gambia, Ghana, Kenya, Nigeria, Rwanda, Somalia, South Africa, Sudan, Uganda, Zambia;

Asia: Afghanistan, Azerbaijan, Kazakhstan, China, Kyrgyzstan, Myanmar, Nepal, Pakistan, Sri Lanka;

Other or unknown: Mexico, unknown.

^b^Some patients had two or more different ESBL-PE, CPE or MRSA strains.

*This designation is without prejudice to positions on status, and is in line with United Nations Security Council Resolution 1244/99 and the International Court of Justice Opinion on the Kosovo Declaration of Independence.

**Table 4:** The number of carriers of multidrug-resistant bacteria among asylum seekers and refugees admitted to Helsinki University Hospital, as presented by five most common countries of origin^a^, Finland, January 2010–August 2017 (n = 447 patients)

Patient attributes	Iraq n = 208		Afghanistan n = 46			Syria n = 43			Somalia n = 31		Nigeria n = 16			Other or unknown n = 103		Total n = 447^b^	
	n	%	n	%		n		%	n	%	n		%	n	%	n	%
**Any MDR bacteria (%)**	**119**	**57.2**	**16**	**34.8**		**24**		**55.8**	**8**	**25.8**	**2**		**12.5**	**32**	**31.1**	**201**	**45.0**
MRSA	65	31.3	5	10.9		9		20.9	4	12.9	1		6.3	11	10.7	95	21.3
ESBL-PE	84	40.4	12	26.1		21		48.8	6	19.4	1		6.3	23	22.3	147	32.9
VRE	0	0.0	0	0.0		0		0.0	0	0.0	0		0.0	0	0.0	0	0.0
MRAB	1	0.5	0	0.0		0		0.0	0	0.0	0		0.0	1	1.0	2	0.4
MRPA	1	0.5	0	0.0		0		0.0	0	0.0	0		0.0	1	1.0	2	0.4
CPE	2	1.0	0	0.0		1		2.3	0	0.0	0		0.0	0	0.0	3	0.7
	**n**	**%**	**n**	**%**		**n**		**%**	**n**	**%**	**n**		**%**	**n**	**%**	**n**	**%**
Multiple MDR bacteria ≥ 2 classes or strains of MDR bacteria	41	19.7	1	2.2		7		16.3	3	9.7	0		0.0	4	3.9	56	12.5
Clinical infection with MDR bacteria	5	2.4	1	2.2		3		7.0	1	3.2	0		0.0	0	0.0	10	2.2
Proportion of MDR bacteria carriers with clinical MDR bacterial infection (%)	4.2		6.3			12.5			12.5		0			0		5.0	
The most common MRSA *spa*-types^c^	*spa*-type	n	*spa*-type		n	*spa*-type	n		*spa*-type	n	*spa*-type	n		*spa*-type	n	*spa*-type	n
	t304 t386 t223 t044 t690 t991	17 8 6 4 3 3	t021 t223 t363 t790 t6238		1 1 1 1 1	t127 t852 t037 t267 t346	2 2 1 1 1		t127 t1784 t8154	1 1 1	t1931	1		t304 t786 t044 t223 t267 t386	2 2 1 1 1 1	t304 t386 t223 t044 t127	19 9 8 5 4

CPE: carbapenemase-producing Enterobacteriaceae; ESBL-PE: extended-spectrum beta-lactamase-producing Enterobacteriaceae; MDR: multidrug-resistant; MRAB: Multiresistant *Acinetobacter baumannii*; MRPA: multiresistant *Pseudomonas aeruginosa*; MRSA: meticillin-resistant *Staphylococcus aureus*; *spa*: staphylococcal protein A; VRE: vancomycin-resistant *Enterococcus*.

^a^In total, the study population originated in 41 different countries.

^b^Some patients had two or more different ESBL-PE, CPE or MRSA strains.

^c^The *spa*-types of some MRSA samples were not available.

### Infections with multidrug-resistant bacteria

Ten of the MDR bacterial carriers (5.0%; 10/201) had a clinical MDR bacterial infection verified by culture, with wound infections and urinary tract infections (UTI) as the most common manifestations. In six cases (60.0%; 6/10) the agent was identified as ESBL-PE, and in four (40.0%; 4/10) as MRSA; 4.1% (6/147) of the ESBL-PE and 4.2% (4/95) of the MRSA carriers presented with a clinical infection. Among patients with a clinical infection as primary diagnosis, 10.8% (9/83) had an MDR bacterial infection.

UTIs were caused by ESBL *E. coli* (one patient) or ESBL *Klebsiella pneumoniae* (one patient). As for wound infections, MRSA was cultured in four cases (one with osteomyelitis) and ESBL *Enterobacter cloacae* in one. One patient with a wound infection had ESBL *Proteus mirabilis* detected both in bacterial culture of wound tissue and blood. Furthermore, there was one patient with a finding of ESBL Shigella, and in one case ESBL *E. coli* was cultured from perianal abscess. No deaths were directly attributed to infections with MDR bacteria.

### Meticillin-resistant *Staphylococcus aureus* findings

The frequency of MRSA findings was 21.3% (95/447) (Table 3). The highest rates were seen among patients coming from Iraq (31.3%; 65/208) and Syria (20.9%; 9/43), followed by those from Somalia (12.9; 4/31) and Afghanistan (10.9%; 5/46) (Table 4). The most common Staphylococcal protein A (*spa*)-types of MRSA isolates were t304 (24.4%), t386 (11.5%), t223 (10.3%), t044 (6.4%), and t127 (6.4%). *Spa*-types seemed to differ by country of origin (Table 4). The median times from arrival in Finland to first sample among MRSA-carriers and non-carriers were 97 and 51 days, respectively (Table 5); the difference was not statistically significant.

**Table 5:** The results of univariate and multivariable risk factor analyses of extended-spectrum beta-lactamase-producing Enterobacteriaceae carriage among asylum seekers and refugees admitted to Helsinki University Hospital, Finland, January 2010–August 2017 (n = 447)

Risk factor	Patients (n = 447)	ESBL-PE carriers (n = 147)		Non-carriers (n = 300)		OR (95% Cl) in univariate analysis	p value in univariate analysis	AOR (95% CI) in multivariable analysis^a^	p value in multivariable analysis
	n	n	%	n	%				
**Sex**									
Female	237	68	28.7	169	71.3	1.0	NA	NA	NA
Male	210	79	37.6	131	62.4	1.5 (1.0–2.2)	0.045	NA	NA
**Age group (years)**							**< 0.001**		
0–5	54	30	55.6	24	44.4	1.0	NA	1.0	NA
6–15	53	16	30.2	37	69.8	0.3 (0.2–0.8)^b^	0.009	0.2 (0.1–0.5)^b^	0.001
16–25	121	30	24.8	91	75.2	0.3 (0.1–0.5)^b^	< 0.001	0.3 (0.2–0.7)^b^	0.003
26–35	152	44	28.9	108	71.1	0.3 (0.2–0.6)^b^	< 0.001	0.4 (0.2–0.8)^b^	0.010
> 35	67	27	40.3	40	59.7	0.5 (0.3–1.1)^b^	0.096	0.5 (0.2–1.2)^b^	0.123
**Geographical region^c^**							**< 0.001**		
Europe	26	3	11.5	23	88.5	1.0	NA	1.0	NA
North Africa and Middle East	268	111	41.4	157	58.6	5.4 (1.6–18.5)^d^	0.007	7.7 (2.1–28.1)^d^	0.002
Sub-Saharan Africa	86	13	15.1	73	84.9	1.4 (0.4–5.2)^d^	0.649	1.4 (0.4–5.9)^d^	0.617
Asia	57	18	31.6	39	68.4	3.5 (0.9–13.3)^d^	0.062	5.9 (1.4–24.1)^d^	0.014
Other or unknown	10	2	20.0	8	80.0	1.9 (0.3–13.6)^d^	0.516	7.0 (0.8–64.9)^d^	0.086
**Prior hospitalisation abroad**									
No or not specified	367	100	27.2	267	72.8	1.0	NA	1.0	NA
Yes	80	47	58.8	33	41.3	3.8 (2.3–6.3)	< 0.001	4.1 (2.3–7.4)	< 0.001
**Prior invasive procedure abroad**									
No or not specified	398	118	29.6	280	70.4	1.0	NA	NA	NA
Yes	49	29	59.2	20	40.8	3.4 (1.9–6.3)	< 0.001	NA	NA
**Prior ICU care abroad**									
No or not specified	429	134	31.2	295	68.8	1.0	NA	NA	NA
Yes	18	13	72.2	5	27.8	5.7 (2.0–16.4)	0.001	NA	NA
**Time from arrival to first sample (days, median)^e^**	**59**	**38**		**83**		**0.91 (0.85–0.96)^e^**	**0.002**	**0.91 (0.86–0.97)**	**0.002**
**Charlson Comorbidity Index (points)**							**0.063**		
0	417	133	31.9	284	68.1	1.0	NA	NA	NA
1	6	1	16.7	5	83.3	0.4 (0.05–3.7)^f^	0.439	NA	NA
2–3	20	13	65.0	7	35.0	4.0 (1.5–10.2)^f^	0.004	NA	NA
4–5	2	0	0	2	100	NA	NA	NA	NA
> 5	2	0	0	2	100	NA	NA	NA	NA

AOR: adjusted odds ratio; CI: confidence interval; ESBL-PE: extended-spectrum beta-lactamase-producing Enterobacteriaceae; ICU: intensive care unit; NA: not applicable; OR: odds ratio; SD: standard deviation.

^a^When selecting the variables for the multivariable model with binary logistic regression, the Akaike information criteria (AIC) was used. Time between arrival and first sample was not known for all cases; missing values were taken into account by multiple imputations, assuming that data were missing at random.

^b^Compared with the youngest age group.

^c^Patients’ countries of origin in the various geographical regions:

Europe: Albania, Georgia, Greece, Kosovo*, Russia, Serbia, Ukraine;

North Africa and Middle East: Iraq, Iran, Jordan, Kuwait, Lebanon, Morocco, Syria, Turkey, Yemen;

Sub-Saharan Africa: Burundi, Cameroon, Congo, Ethiopia, Eritrea, Gambia, Ghana, Kenya, Nigeria, Rwanda, Somalia, South Africa, Sudan, Uganda, Zambia;

Asia: Afghanistan, Azerbaijan, Kazakhstan, China, Kyrgyzstan, Myanmar, Nepal, Pakistan, Sri Lanka.

^d^Compared with Europe.

^e^n = 283; only in 283/447 cases arrival date to Finland was known. Analysed as a continuous variable, OR and adjusted OR given per 30 days.

^f^Compared with those with 0 points.

*This designation is without prejudice to positions on status, and is in line with United Nations Security Council Resolution 1244/99 and the International Court of Justice Opinion on the Kosovo Declaration of Independence.

### Multiresistant Gram-negative bacteria findings

CPE strains (*K. pneumoniae, E. coli, Acinetobacter baumannii,* and *Proteus mirabilis*) were found in the samples of two patients from Iraq and one from Syria; both Iraqis had multiple CPE strains. MRPA was recorded for two patients, one Russian, the other Iraqi. One refugee from Cameroon and one from Iraq were screened positive for MRAB.

One third of the patients (32.9%; 147/447) were colonised by ESBL-PE, with the highest frequency seen among patients of North African and Middle Eastern origin (41.4%; 111/268). Nearly half of the Syrians (48.8%; 21/43) were ESBL-PE carriers, while Iraqis and Afghans had carriage rates of 40.4% (84/208) and 26.1% (12/46), respectively (Table 4). Most ESBL-PE strains were *E. coli* (90.2%), followed by *K. pneumoniae* (6.1%), *Proteus mirabilis* (2.4%) and *Enterobacter cloacae* (0.6%). The median time from arrival in Finland to first sample was significantly shorter among ESBL-PE-carriers (38 days) than non-carriers (83 days) (Table 5).

### Extended-spectrum beta-lactamase-producing Enterobacteriaceae with co-resistance to other antimicrobials

Co-resistance to other antibiotics proved common among the ESBL strains: two thirds (62.2%; 102/164) of the ESBL-PE isolates showed decreased sensitivity to co-trimoxazole and more than one third were co-resistant to levofloxacin (40.2%; 66/164) or tobramycin (34.1%; 56/164). Resistance to levofloxacin was associated with co-resistance to tobramycin (OR 6.0, 95% confidence intervals (CI): 3.0–12.2) but not co-trimoxazole (OR 1.7, 95% CI: 0.9–3.3) in univariate analysis. No statistical differences were seen in co-resistance between the various geographical regions.

### Risk factor analysis of meticillin-resistant *Staphylococcus aureus* colonisation

In multivariable analysis, geographical region and prior invasive procedure outside the Nordic countries were found to be independent risk factors of MRSA colonisation. No other risk factors were identified. (Table 6)

**Table 6:** The results of univariate and multivariable risk factor analyses of meticillin-resistant *Staphylococcus aureus* colonisation among asylum seekers and refugees admitted to Helsinki University Hospital, Finland, January 2010 to August 2017 (n = 447)

Risk Factor	Patients (n = 447)	MRSA carriers (n = 95)		Non-carriers (n = 352)		OR (95% Cl) in univariate analysis	p value in univariate analysis	AOR (95% CI) in multivariable analysis^a^	p value in multivariable analysis
	n	n	%	n	%				
**Sex**									
Female	237	47	19.8	190	80.2	1.0	NA	NA	NA
Male	210	48	22.9	162	77.1	1.2 (0.8–1.9)	0.435	NA	NA
**Age group (years)**							**0.806**		
0–5	54	13	24.1	41	75.9	1.0	NA	NA	NA
6–15	53	11	20.8	42	79.2	0.8 (0.3–2.1)^b^	0.681	NA	NA
16–25	121	22	18.2	99	81.8	0.7 (0.3–2.1)^b^	0.369	NA	NA
26–35	152	36	23.7	116	76.3	1.0 (0.5–2.0)^b^	0.954	NA	NA
> 35	67	13	19.4	54	80.6	0.8 (0.3–1.8)^b^	0.535	NA	NA
**Geographical region^c^**							**0.002**		
Europe	26	2	7.7	24	92.3	1.0	NA	1.0	NA
North Africa and Middle East	268	75	27.6	197	72.4	4.7 (1.1–20.2)^d^	0.040	4.5 (1.0–19.7)^d^	0.044
Sub-Saharan Africa	86	11	13.4	71	86.6	1.8 (0.4–8.5)^d^	0.482	1.7 (0.4–8.4)^d^	0.500
Asia	57	5	8.8	52	91.2	1.2 (0.2–6.4)^d^	0.870	1.2 (0.2–6.4)^d^	0.864
Other or unknown	10	2	20.0	8	80.0	3.0 (0.4–24.9)^d^	0.309	3.2 (0.4–26.9)^d^	0.279
**Prior hospitalisation abroad**									
No or not specified	367	72	19.6	295	80.4	1.0	NA	NA	NA
Yes	80	23	28.8	57	71.3	1.7 (1.0–2.9)	0.072	NA	NA
**Prior invasive procedure abroad**									
No or not specified	398	78	19.6	320	80.4	1.0	NA	1.0	NA
Yes	49	17	34.7	32	65.3	2.2 (1.2–4.1)	0.017	2.0 (1.1–3.9)	0.033
**Prior ICU care abroad**									
No or not specified	429	89	20.7	340	79.3	1.0	NA	NA	NA
Yes	18	6	33.3	12	66.7	1.9 (0.7–5.2)	0.208	NA	NA
**Time from arrival to first sample (days, median)^e^**	**59**	**97**		**51**		**1.05 (0.99–1.10)^e^**	**0.079**	**NA**	**NA**
**Charlson comorbidity index (points)**							**0.626**		
0	417	93	22.3	324	77.7	1.0	NA	NA	NA
1	6	1	16.7	5	83.3	0.7 (0.1–6.0)^f^	0.743	NA	NA
2–3	20	1	5.0	19	95.0	0.1 (0.02–1.4)^f^	0.100	NA	NA
4–5	2	0	0	2	100	NA	NA	NA	NA
> 5	2	0	0	2	100	NA	NA	NA	NA

AOR: adjusted odds ratio; CI: confidence interval; ICU: intensive care unit; MRSA: meticillin-resistant *Staphylococcus aureus*; NA: not applicable; OR: odds ratio; SD: standard deviation.

^a^When selecting the variables for the multivariable model with binary logistic regression, the Akaike information criteria (AIC) was used. Time between arrival and first sample was not known for all cases; missing values were taken into account by multiple imputations, assuming that data were missing at random.

^b^Compared with the youngest age group.

^c^Patients’ countries of origin in the various geographical regions:

Europe: Albania, Georgia, Greece, Kosovo*, Russia, Serbia, Ukraine;

North Africa and Middle East: Iraq, Iran, Jordan, Kuwait, Lebanon, Morocco, Syria, Turkey, Yemen;

Sub-Saharan Africa: Burundi, Cameroon, Congo, Ethiopia, Eritrea, Gambia, Ghana, Kenya, Nigeria, Rwanda, Somalia, South Africa, Sudan, Uganda, Zambia;

Asia: Afghanistan, Azerbaijan, Kazakhstan, China, Kyrgyzstan, Myanmar, Nepal, Pakistan, Sri Lanka.

^d^Compared with Europe.

^e^n = 283, only in 283/447 cases arrival date to Finland was known. Analysed as a continuous variable, OR and adjusted OR given per 30 days.

^f^Compared with those with 0 points.

*This designation is without prejudice to positions on status, and is in line with United Nations Security Council Resolution 1244/99 and the International Court of Justice Opinion on the Kosovo Declaration of Independence.

### Risk factor analysis of extended-spectrum beta-lactamase-producing Enterobacteriaceae colonisation

We identified by univariate analysis the following factors as predisposing to ESBL-PE: young age, geographical region, short time from arrival to first sample, prior hospitalisation, prior invasive procedure abroad, and prior ICU care (Table 5). The final multivariable analysis revealed geographical region of origin, age under 6 years, short time from arrival to first sample and prior hospitalisation abroad as independent risk factors for colonisation. As for the geographical region of origin, the ESBL-PE colonisation rates were significantly higher among North African, Middle Eastern and Asian patients than Europeans (Table 5). Of factors with p value under 0.2 in univariate analysis, male sex, prior invasive procedure abroad, prior ICU care, and general health (CCI score) were eliminated from the final model on the basis of the AIC.

## Discussion

Data remain scarce on AMR carriage rates in refugees and migrants [24]. The present investigation reveals MDR bacterial colonisation and a high prevalence of MRSA of 21.3% among asylum seekers and refugees seeking healthcare at a tertiary hospital in Finland.

Although focusing on AMR, we also collected general data on refugees and migrants and causes of their hospitalisation. Although 79% of the asylum seekers who have arrived in Finland since 2015 have been male [34], in our data no clear sex difference in hospital admission was seen; the discrepancy can probably be explained by our high number of obstetric hospitalisations. Overall, our patients were in good general health; the greatest number of visits (29.8%) were reported for the specialty of obstetrics, while infectious diseases accounted for fewer than one in five cases. Respiratory, skin, and urinary tract infections were the most common causes among infectious diseases. Obviously, these are also common causes of hospitalisation among the host population. The proportion of minors corresponded to that of refugee and migrant minors arriving in Finland (24.9%) [35].

### Level of colonisation by any multidrug-resistant bacteria

Nearly half of the patients were colonised by MDR bacteria, a finding consistent with recent research focusing on refugees admitted to hospitals [36-38]. In Germany, Tenenbaum et al. reported an MDR bacterial carriage rate of 33.8% (110/325) in a paediatric population in 2015 to 2016 [36], and Reinheimer et al. rates of 52.1% (61/117) in 2015 to 2016 and 66.4% (95/143) in 2015 in two studies with both adult and paediatric patients [37,38]. The various countries of birth may account for the differences: in the previous studies, most of the patients were Syrians and Afghans, whereas in ours, nearly half were Iraqis. The high overall rate of MDR bacterial colonisation among refugees and asylum seekers justifies infection control measures, according with rates of 55.2% demonstrated in another group routinely subjected to isolation at hospitals in low-prevalence countries, namely travellers with a recent history of hospitalisation in the (sub)tropics [39].

As we expected, the clinical ESBL-PE infections generally proved to be UTIs; MDR bacterial wound infections were mostly attributed to MRSA. Data on the frequency of MDR bacterial carriers developing an infection with the MDR bacterial strain are scarce. Among returning travellers treated on an infectious and tropical diseases ward in France from 2012 to 2013, Epelboin et al. [40] found that one in five MRGN bacterial carriers identified (21.7%, 5/23) also had a clinical MRGN bacterial infection, albeit in a very small sample. The difference between our rates and those of Epelboin et al. (5.0% vs 21.7%, respectively) can probably be explained by two points related to the selection of population. Firstly, our data included a high proportion of healthy obstetric patients with no MDR bacterial infections. Secondly, in the investigation by Epelboin all patients had a clinical infection, while ours looked retrospectively at all patients, admitted for any reason, who at admission to a tertiary hospital had been sampled for both MRSA and MRGN bacteria, and whom we could identify as asylum seekers and refugees from their patient records. Indeed, of our patients who had infectious disease as primary diagnosis, 10.8% had an MDR bacterial infection. In our previous study looking at 1,122 Finnish patients hospitalised abroad for diverse reasons, we found that 11.4% (38/333) of those colonised with MDR bacteria had an infection with the same MDR bacteria [39].

### Colonisation by MRSA

A major finding emerging from our investigation was that MRSA carriage in asylum seekers and refugees is considerably more common than expected. This proportion significantly exceeds those of German studies that have reported rates from 5.6% to 9.8% (8/143 and 32/325) [37,41], and rates of 15.7% recorded at Swiss asylum seeker reception centres [42]. MRSA carriage also proved more prevalent in the study population than among regular travellers hospitalised in the (sub)tropics: Khawaja et al. reported 6.6% (25/377) for 2010–2013 [39], Kaspar et al. 2% for 2012–2013 [43], Nemeth et al. 1.2% for 2009–2011 [44], and Birgand et al. 3.8% for 2012–2013 [45]. This relevant and valuable finding should be taken heed of when drawing up infection control guidelines for hospital admission of refugees and asylum seekers.

An analysis of *spa*-types revealed that the MRSA strains differ from those most commonly seen in clinical samples in Finland (t008, t172, t067) [46]. Clear differences were found between those with different countries of origin, suggesting that the strains had not originated in Finland but that refugees had been MRSA carriers on arrival. The median time from arrival in Finland to first sample was almost twice as long among MRSA carriers than among non-carriers. A tendency was seen for MRSA rates to grow as their stay in Finland lengthened, but the difference was not statistically significant (Table 6).

### Colonisation by multiresistant bacteria

The rising number of CPEs has aroused great concern in Europe [47]. CPE findings have been reported for 0–2.1% of refugee patients [37,41,48]. Likewise, our study found the CPE rates to be low. Of course, even at these levels, they exceed the background rate in Finland [49]; special attention is warranted, since they may rise further, running parallel to increasing AMR prevalence in the various countries of origin.

As for MRAB and MRPA colonisation, two cases of each were identified; none with VRE were found, a valuable piece of information for professionals planning infection control measures in hospitals.

ESBL-PE were the most commonly recorded MDR bacteria. Detected among one third of the refugees and migrants, the rates remained lower than in two studies by Reinheimer et al. [37,38] in Germany, reporting 52.1% (61/117) and 58.7% (84/143) prevalence, yet accorded with rates identified in samples from unaccompanied refugee minors in Frankfurt in 2015 (35.3%, 42/119) [50]. The colonisation rates among refugees resembled those reported for regular travellers (20–70%) [51,52], the figures probably reflecting country-related background colonisation rates. Traveller studies show significant differences depending on destination [51,52]; the highest numbers are seen among visitors to major risk regions such as the Indian subcontinent.

It appears, however, that the initial ESBL-PE rates of asylum seekers and refugees on arrival in Finland may actually have been higher than recorded here: those with longer time since arrival had a lower ESBL-PE carriage frequency than those sampled soon after immigration. Indeed, recent follow-up studies show that travellers’ ESBL-PE carriage tends to be transient and detectable only for a few months after return [15,53]. While the acquisition rates reported in traveller studies are based on samples collected soon after travellers’ return, in the present study the median time from arrival to sampling was approximately 8 weeks. Further research is needed into carriage duration among refugees and migrants or other people with a recent history of long-term exposure to MDR bacteria.

A substantial proportion of our ESBL-PE isolates proved co-resistant to levofloxacin, tobramycin or co-trimoxazole, which accords with the results of studies exploring colonisation among travellers [4,9,11,12]. Co-resistance to levofloxacin correlated with resistance to tobramycin but not co-trimoxazole. This finding may be related to a genetic linkage between the resistance mechanisms of the first two [54,55].

### Risk factor analysis

To identify potential risk factors of MRSA and ESBL-PE colonisation, we conducted univariate and multivariable analysis of the items derived from the patient records. To our knowledge, until now, risk factor analyses have not been included in refugee/migrant studies [36-38,41,48,50].

As independent risk factors of MRSA colonisation, we recognised geographical region and prior invasive procedure outside the Nordic countries. The latter accords with previous studies showing prior healthcare contact to be a risk factor of MRSA colonisation at hospital admission [56,57]. In our research, the highest risk of MRSA colonisation was seen among patients from North Africa and Middle East, which is in line with an investigation by Stenhem et al. analysing imported MRSA cases in Sweden from 2000 to 2003 [58]. Some of the other risk factors of MRSA colonisation such as prior infections or antibiotic treatments, occupation (e.g. healthcare worker) or contact with livestock could not be covered, due to the retrospective nature of our study [57].

For ESBL-PE colonisation, the final multivariable analyses revealed as independent risk factors geographical region, young age (< 6 years old), short time from arrival to first sample, and prior hospitalisation outside the Nordic countries. Geographical region has been identified in virtually all traveller studies analysing ESBL-PE risk factors [5,12-14,16,52]. The highest risk has been linked with Asia and the Middle East [5-7,9-18], a finding that agrees with our results. Likewise, prior hospitalisation has been established as a risk factor for MDR bacterial carriage among travellers [59]. One previous result contradictory to ours showed a correlation between young age and lower rates of ESBL-PE colonisation [13]. Indeed, refugee children and travelling minors cannot be regarded as comparable: tourist children’s exposure to food/drink contaminated with intestinal microbes is of shorter duration, and, moreover, trying to avoid diarrhoea, their parents probably select less risky food for them.

Traveller studies have also identified a number of other risk factors, such as occurrence of diarrhoea [5,6,9,10,13-15] and use of antibiotics [6,13,15-17]. Unfortunately, these predisposing factors could not be included in our analyses since such data could not be consistently drawn from our patient records.

### Limitations

Due to the retrospective design of our study, the data were limited to those available in patient records. Some relevant factors such as antibiotic use could not be analysed. Information concerning the itineraries of the refugees and asylum seekers was lacking, and dates of arrival in Finland were recorded for only 63.3% of the patients. The research was conducted in a tertiary hospital which is reflected in the selection of the patients. Females were over-represented in our study population (53.0%), probably because of the large proportion of pregnancy-related hospital visits. After the beginning of 2015 only 21.2% of all asylum seekers arriving in Finland were female [60].

### Conclusions

Our study shows considerable carriage rates for MDR bacteria among refugees and asylum seekers admitted to a tertiary hospital in Finland. The data suggest that these patients should be considered a risk group that requires both screening of MDR bacteria and infection control measures at entry to hospitals in low-prevalence countries. In particular the refugee and migrant population’s considerable MRSA colonisation rate warrants attention in healthcare settings.

## Note

*This designation is without prejudice to positions on status, and is in line with United Nations Security Council Resolution 1244/99 and the International Court of Justice Opinion on the Kosovo Declaration of Independence.
